# Influence of intermetallic phase (TiFe) on the microstructural evolution and mechanical properties of as-cast and quenched Ti–Mo–Fe alloys

**DOI:** 10.1038/s41598-024-60894-x

**Published:** 2024-05-07

**Authors:** Nthabiseng Moshokoa, Elizabeth Makhatha, Lerato Raganya, Washington Makoana, Hasani Chauke, Ramogohlo Diale, Maje Phasha

**Affiliations:** 1 Department of Metallurgy, School of Mining and Metallurgy and Chemical Engineering, University of Johannesburg, Doornfontein Campus, Johannesburg, South Africa; 2 Advance Materials Engineering, Manufacturing Cluster, Council for Scientific and Industrial Research, Meiring Naude Road, Brummeria, Pretoria 0184 South Africa; 3 National Laser Center, Council for Scientific and Industrial Research, Meiring Naude Road, Brummeria, Pretoria 0184 South Africa; 4 Materials Modelling Center, University of Limpopo, Private Bag X1106, Sovenga, 0727 South Africa; 5 Advanced Materials Division, Physical Metallurgy Group, Mintek, 200 Malibongwe Drive, Randburg, 2125 South Africa

**Keywords:** Ti–Mo–Fe, Microstructure, Mechanical properties, Intermetallic phase, Engineering, Materials science

## Abstract

This study presents the phase analysis, microstructural characteristics, and mechanical property evaluation of the as-cast and quenched Ti–15Mo–xFe alloys with high iron content ranging from 4 to 12 weight percent. All the four alloys were produced in a vacuum-arc melting furnace. Heat treatment in the form of solution treatment was performed in a muffle furnace at a temperature of 1100 °C, with 1-h holding time and the samples were rapidly quenched in ice-brine. X-ray diffractometer (XRD) was used to analyses the phases present in each alloy whereas the optical microscope (OM) was employed to track the microstructural evolution and percentage porosity. The mechanical properties of the alloys were evaluated using a tensile test and compression test method while the micro-Vickers hardness measurements were conducted to evaluate hardness of the alloys. The XRD patterns of as-cast showed peaks belonging to the β and α″ phases and intermetallic B2 TiFe phases. The as quenched XRD peaks illustrated β phase only and Fe·Ti·O_2_ phases. The as-cast OM micrographs revealed equiaxed β grains, substructures, dendritic structure, and pores forming around the grain boundaries. The quenched OM showed only β equiaxed grains with pores throughout the grain boundaries. The tensile properties such as ultimate tensile strength (UTS) and elastic modulus (*E*) of as-cast TMF0 were 264 MPa and 79 GPa respectively and these properties changed upon quenching to 411 MPa and 66 GPa respectively. The elastic modulus of TMF1 in as-cast condition was 74 GPa. The UTS and *E* of TMF1, TMF2, and TMF3 in as-cast and quenched conditions were not recorded due to the fragility of the samples that failed prior to yielding any useful data. The compressive strength in as-cast and in quenched condition decreased with an increase in Fe content. The micro-Vickers hardness in as-cast and quenched conditions showed a similar trend with hardness increasing slightly upon quenching for TMF0, TMF1, and TMF3 alloys but slightly decreased in the case of TMF2. The fracture surfaces of all the as-cast and quenched alloys were comprised of ductile and brittle fracture.

## Introduction

Metallic materials are vastly deployed in load bearing structures of the human body such as hip and knee implants because of their exceptional properties such as high mechanical strength, fatigue resistance and ease of machining. In such biomedical applications, it is important for the biomaterial to possess long term durability when implanted in the human body to support and stabilize the bone and joints^1^. Presently, at least two-thirds of implants are produced from metallic biomaterials such as stainless steel, cobalt-chromium alloys, and titanium and its alloys^2^. Amongst the metallic biomaterials, titanium (Ti) and its alloys with appealing properties such as high specific strength, high corrosion resistance, low elastic modulus, and excellent biocompatibility are increasingly finding applications in replacing or repairing failed hard tissues such as artificial hip joints, dental implants, etc.^3^. Within the Ti and its alloys group, Ti6Al4V alloy is the most used in the fabrication of orthopedic implants because of properties such as high strength-to-weight ratio, good corrosion resistance, and low elastic modulus as compared to other metallic biomaterials such as nickel (Ni) alloys, cobalt-chromium (Co-Cr) alloys and stainless steel^4^. Nevertheless, Ti6Al4V material poses two major health scares. Firstly, the release of aluminium (Al) and vanadium (V) ions into the human blood, an effect associated with neurological disorders such as Osteomalacia, and anaemia^5^. Secondly, the mismatch in elastic modulus between the implant material (110 GPa) and that of the human bone (10–40 GPa), resulting in challenges such as absorption, stress shielding, and atrophy^6^. The above drawbacks stimulated research into design and development of β-Ti type alloys with non-toxic elements such as niobium (Nb), tantalum (Ta), zirconium (Zr), molybdenum (Mo), and tin (Sn) ect, which are biocompatible, with moderate strength and the lowest modulus as compared to the Ti6Al4V alloy. The candidacy alloy are subjected to unique requirements such as biocompatibility which is essential for the interaction with human tissues in the biological environment, the mechanical properties such as moderate strength and low elastic modulus to avoid the stress shielding effects, the alloy must have high resistance to corrosion and wear resistance^1^. Current developed alloys to be used as metallic biomaterials include: Ti–13Nb–13Zr^7^, Ti–12Mo–6Zr–2Fe (TMZF)^8^, Ti–15Mo^9^, Ti–Nb_17_Ta_6_O_1_ (TNTO)^10^, Ti–29Nb–13Ta–4.6Zr (TNTZ)^11^, Ti–35Nb–2Ta–3Zr^12^ and Ti2448^13^ alloys which demonstrated low elastic modulus, moderate strength, better corrosion properties when studied under different processing techniques. However, most of these alloys are composed of high-cost and rare elements such as Ta, Nb, and Zr in which they lead to high cost of raw materials. Consequently, recent developments are focusing on developing new low-cost β-type Ti alloys for biomedical and structural applications which consist of low-cost alloying elements such as iron (Fe), manganese (Mn), etc. Fe is selected as an alloying element to Mo because it is cheaper, it is a strong β stabilizer, solution-strengthening element, and capable of effectively decreasing the melting point of Ti alloy^14^. An addition of only 4wt.% Fe is required to retain 100% β phase after quenching to room temperature, therefore Fe could be used to replace other β stabilizing elements at a lower cost^15^. However, restricted use of Fe is generally due to processing issues associated with the melting process which can affect the quality of the resulting alloys^16^. For example, segregation of Fe during vacuum arc remelting can lead to localized regions of high β stability known as β flecks, which cause worse-than-expected mechanical performance^17^. Therefore, one of the basic design strategy is that the single β-Ti structure should be retained by rapid cooling and by selecting the Fe content lower than the Ti–Fe eutectoid point (15 at% Fe) to avoid the formation of brittle Ti–Fe phase. Several studies have been reported on the addition of Fe into Ti and metastable β Ti alloys. For example β Ti–Fe alloys exhibiting a eutectic transformation (β > β + TiFe) have previously demonstrated extremely high strengths^18^. A high concentration of Fe (> 20 pct) is required for the β > β + TiFe transformation to occur because of the high solubility of Fe in this β phase. Contieri et al. reported the compressive behavior of a Ti–32.5Fe alloy with a lamellar structure via β > β + TiFe. As the interphase spacing between β and TiFe decreased from 1.5 to 0.7 μm, the strength increased substantially from 1844 to 3000 MPa^19^. Chaves et al. studied the phase transformation and elastic modulus of Ti–xNb–3Fe (x = 10, 15, 20 and 25 wt%), their study revealed that increasing the Nb content stabilized the β phase and no intermetallic phase was formed^20^. Qi et al. evaluated the tensile and elastic modulus of Ti–6Zr–xFe (x = 4, 5, 6, 7 wt%) alloy. They reported no formation of intermetallic phase of TiFe, however the strength, hardness and elastic modulus increased with an increase in Fe content^21^. Haghighi et al. investigated the effect of Fe on the phase transition, microstructure and mechanical properties of the Ti–11Nb–xFe (x = 1/2, 3.5, 5, 6 & 9 wt%) alloy. the results showed that as the Fe content increased the yield strength, plastic strain and micro-Vickers hardness increased significantly^22^. Recent research work reported increased strength when large amounts of twins formed during plastic deformation by just adding 1 weight percent (wt.%) of Fe to Ti–10Mo and Ti–15Mo alloy^23^. Furthermore, superior mechanical properties, good corrosion resistance as well as outstanding biocompatibility were observed when 2–5 wt.% Fe was added to Ti–10Mo alloy^24^. Ti–Mo–Fe alloys are mostly developed for biomedical applications owing to their properties and their vast results on different processing and characterization technique are been published in literature. However work on the influence of the intermetallic phases (TiFe) due to high addition of Fe (near the eutectic point) in Ti–15Mo alloy processed by arc melting furnace is still lacking. Thus the aim of this study is to investigate the effect of the intermetallic phase (B2 TiFe) on the microstructure and mechanical properties of Ti–15Mo–xFe alloy by addition of high Fe content. Four alloys of Ti–15Mo–xFe (x = 0, 4, 8 & 12) were fabricated by vacuum arc remelting furnace. The cast ingots were heat treated in a muffle furnace at high temperature and quenched in ice-brine. The analyses were comprised of phase evolution, microstructural characteristics, as well as tensile, compression and micro-hardness tests.

## Experimental procedure

### Material preparation

The Ti–15Mo–xFe alloys with Fe content of 0, 4, 8, and 12 wt% here referred to as TMF0, TMF1, TMF2, and TMF3, respectively, were prepared from high-purity metal powders, Ti (99.5%), Mo (99.95%), and Fe (99.9%). All four alloys were produced in a vacuum arc melting furnace with a water-cooled copper hearth. In attempt to avoid contamination, the melting was conducted under an argon atmosphere. The ingots were turned and re-melted at least three times in order to ensure homogeneity. Sectioned as-cast samples were solution treated in a muffle furnace at 1100 °C, with holding time of 1 h and followed by subsequent rapid quenching in ice-brine.

### Phase and microstructural characterisation

X-ray diffractometer (XRD) was used to conduct phase analysis using the Phillips Xpert Pro PANalytical Netherland operated at 45 kV and 40 mA. The Cu Kα radiation with a secondary monochromatic (λ = 0, 1545 nm) was utilized to run the XRD patterns. Diffraction measurements were conducted at room temperature in Bragg-Brenton geometry with a scan of 2θ range of 30°–80° using continuous scanning at a rate of 0.02°. Phases present were identified by matching each characteristic peak with X-Pert High score software. In order to track the microstructural evolution, the surfaces of the as-cast and water-quenched samples were grinded using a series of silicon carbide papers up to 2400 grit paper, followed by mechanical polishing using the polishing cloths embedded with colloidal silica. After final polishing, the samples were etched with a solution consisting of distilled water, hydrofluoric acid, and nitric acid (80:15:5 in volume) for 35 s. Microstructure of the samples was investigated using an optical microscope (OM).

### Mechanical properties

The Micro-Vickers hardness for all the as-cast and water-quenched samples was measured using the Zwick Roell Vickers hardness indenter. The indents were made from a small diamond under the load of 500gf for 10 s. For each sample, 10 indents were made with a 2 mm distance and measured microscopically and finally, their averages were recorded. In addition, the tensile strength, and Young’s modulus were investigated. Tensile specimens with 3 × 4 × 30 mm gauge were prepared by electrical discharge machining. Tensile tests were performed at room temperature using an Instron™ 1342 tensile tester fitted with 50 kN load cells with a constant crosshead speed of 0.5 mm/min. An extensometer was attached to the gauge section of the test specimen and was used to measure the tensile strain. The fracture surfaces of the tensile specimens were analyzed using JOEL JSM-6010 Plus/LAM scanning electron microscope (SEM) at accelerating voltage of 8 kV and all the images were captured at a magnification of 350× (50 μm). Compression test at room temperature were performed on rectangular specimens with dimensions of 4 × 4 × 8mm from each alloy on an Ingstron^TM^ 1342 machine. The test was carried out following the ASTME9 standard where three specimens from each ally were tested/ until failure.

## Results and discussions

### Phase analysis

**Figure 1:**
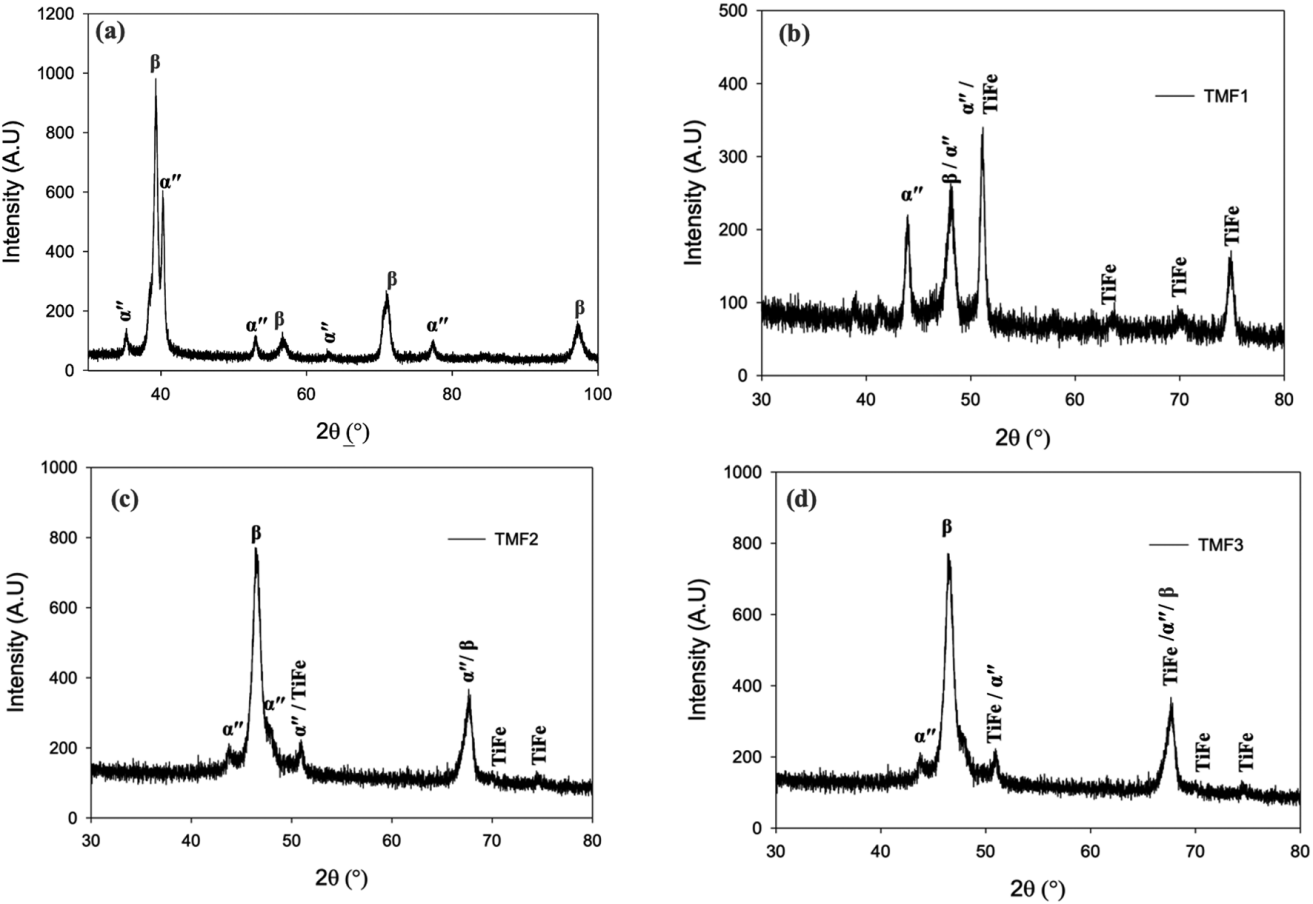
XRD results of as-cast, (**a**) TMF0 (**b**) TMF1, (**c**) TMF2 and (**d**) TMF3 alloys.

**Figure 2:**
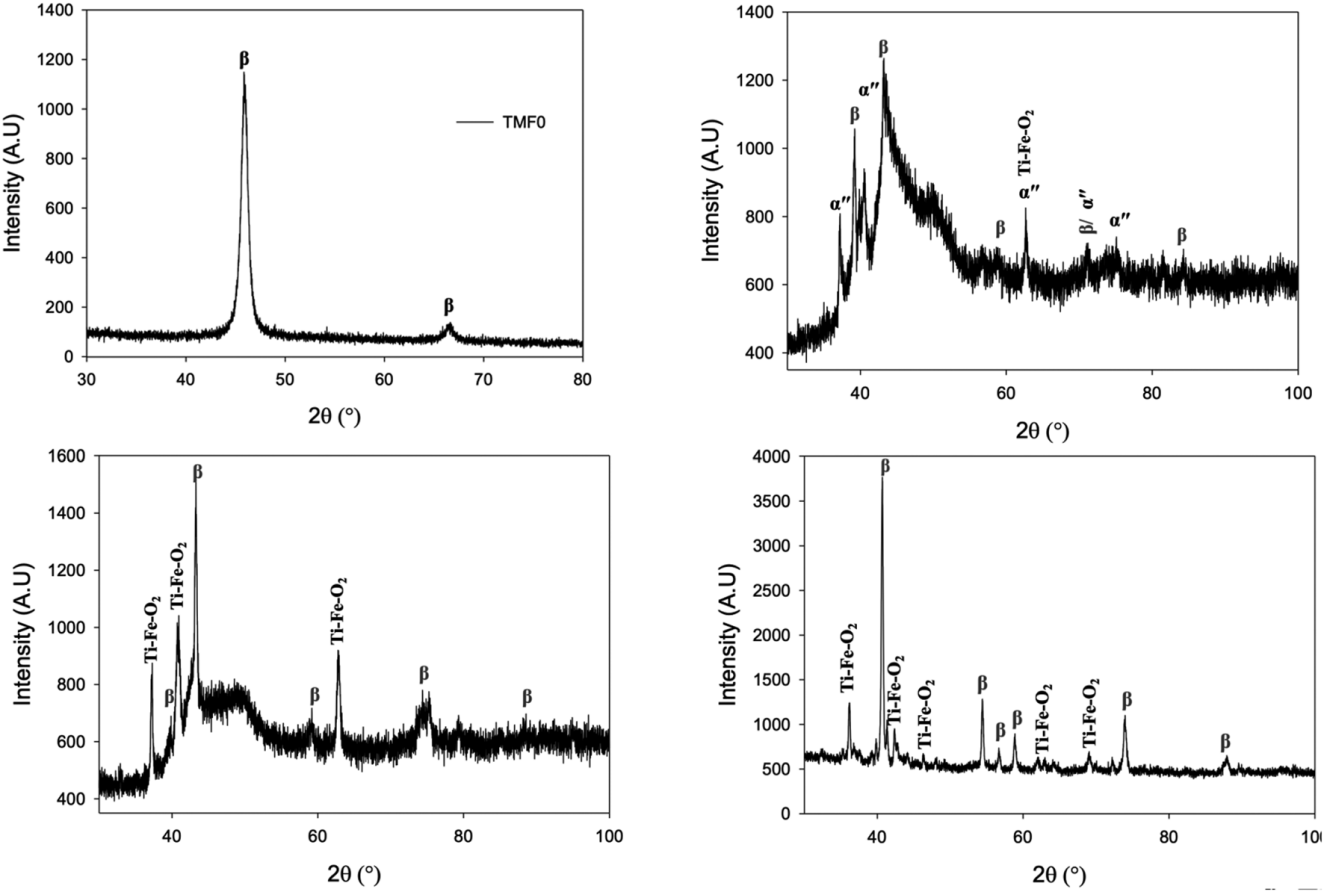
XRD results of quenched (**a**) TMF0, (**b**) TMF1, (·) TMF2 and (**d**) TMF3 alloys.

Presented in Figs. 1 and 2 are the XRD patterns of all considered alloys in the as-cast and quenched condition, respectively. The TMF0 in the as-cast condition show peaks belonging to both bcc (β) phase and orthorhombic martensitic α′′ phase, as shown in Fig. 1a. The quenched sample depicted peaks belonging to only bcc β phase peaks only as observed in Fig. 2a. Presence of other metastable phases such omega and orthorhombic is not detected. Current results imply that the cooling rate experience by the sample when quenched from 1100 °C into ice-brine was fast enough to retain complete (100%) β phase. This heat-treatment process did not afford any atomic displacement enough time to enable diffusionless martensitic transformation to occur. The addition of 4, 8 and 12wt.% Fe into Ti–15Mo, corresponding to TMF1 in Fig. 1b, TMF2 in Fig. 1c, and TMF3 in Fig. 1d, respectively, the XRD patterns of the as-cast samples revealed peaks belonging to β, α′′ and the ordered B2 intermetallic phase belonging to TiFe. Interestingly, the TMF1 alloy demonstrated the highest volume fraction of the intermetallic phase compared to TMF2 and TMF3 alloys. It also worth noting that the fraction of orthorhombic martensitic α′′ phase decreased gradually with an increase in Fe content whereas the volume fraction of the intermetallic B2 phase increased. The presence of the orthorhombic martensitic α′′ phase in TMF1, TMF2 and TMF3 showed that the cooling rate during melting was not sufficient to retain the β phase. At the same time, the presence of the intermetallic phase signified that this cooling rate was slow enough to allow formation and precipitation of B2 TiFe to occur from liquidus phase. It also worth noting that the addition of Fe resulted in a peak shift towards higher 2θ range. This peak shift can be attributed to lattice contraction due to smaller atomic radius of Fe.

The XRD results of quenched TMF1 sample shown in Fig. 2b revealed peaks belonging to both the α′′ and β phases as well as Ti–Fe mixed oxide (Fe·Ti·O_2_). On the other hand, the XRD results of quenched TMF2 and TMF3 shown in Fig. 2c, d, respectively, depicted only peaks belonging to β and the Ti–Fe mixed oxides (Fe·Ti·O_2_) phases. The volume fraction of the mixed oxide phase increased with an increase in Fe content with the TMF3 sample detected to have the most significant amount. The presence of the metal oxides can be ascribed to air exposure when the sample was taken out of the furnace to the quenching bath. As a result of solution treatment, the presence of intermetallic B2 TiFe phase was not detected in the quenched samples. The disappearance of the TiFe phase in the quenched samples suggests that the solution treatment temperature of 1100 °C was sufficient dissolve B2 TiFe since from the Ti–Fe binary phase diagram, the B2 TiFe only starts to dissolve into the β Ti(Fe) matrix at 1085 °C. On contrary, it is also possible that the B2 phase contained some amount of Mo, which would increase the temperature where B2 phase dissolve into the β matrix to above 1100 °C. This would render the B2 phase undissolved, thus suggesting the intermetallic phase to be more prone to oxidation at high temperatures than the β phase, responsible to forming mixed oxide. Therefore further analytical work is still necessary to understand the underlying mechanism. The XRD pattern of TMF0 in quenched condition is analogous to the results described by^15,20,21^ in Ti–15Mo binary alloy. The presence of peaks belonging to the intermetallic B2 structure (TiFe) in the ternary alloys were comparable to XRD peaks reported by^22^ in Ti_65_Fe_35_ binary alloy in as-cast condition and the TiFe was also reported by^23^ in Ti–19Fe–5Sn–1Mo, Ti–17Fe–5Sn–3Mo and Ti–15Fe–5Sn–5Mo alloys. The presence of peaks of orthorhombic martensitic α′′ phase in the ternary alloys in the as-cast condition were also reported in a ternary alloy Ti–7Ta–5Fe alloy reported by^24^ and also in Ti–Zr–4Fe, and Ti–Zr–5Fe alloys investigated by^25^.

### Microstructural evolution

**Figure 3:**
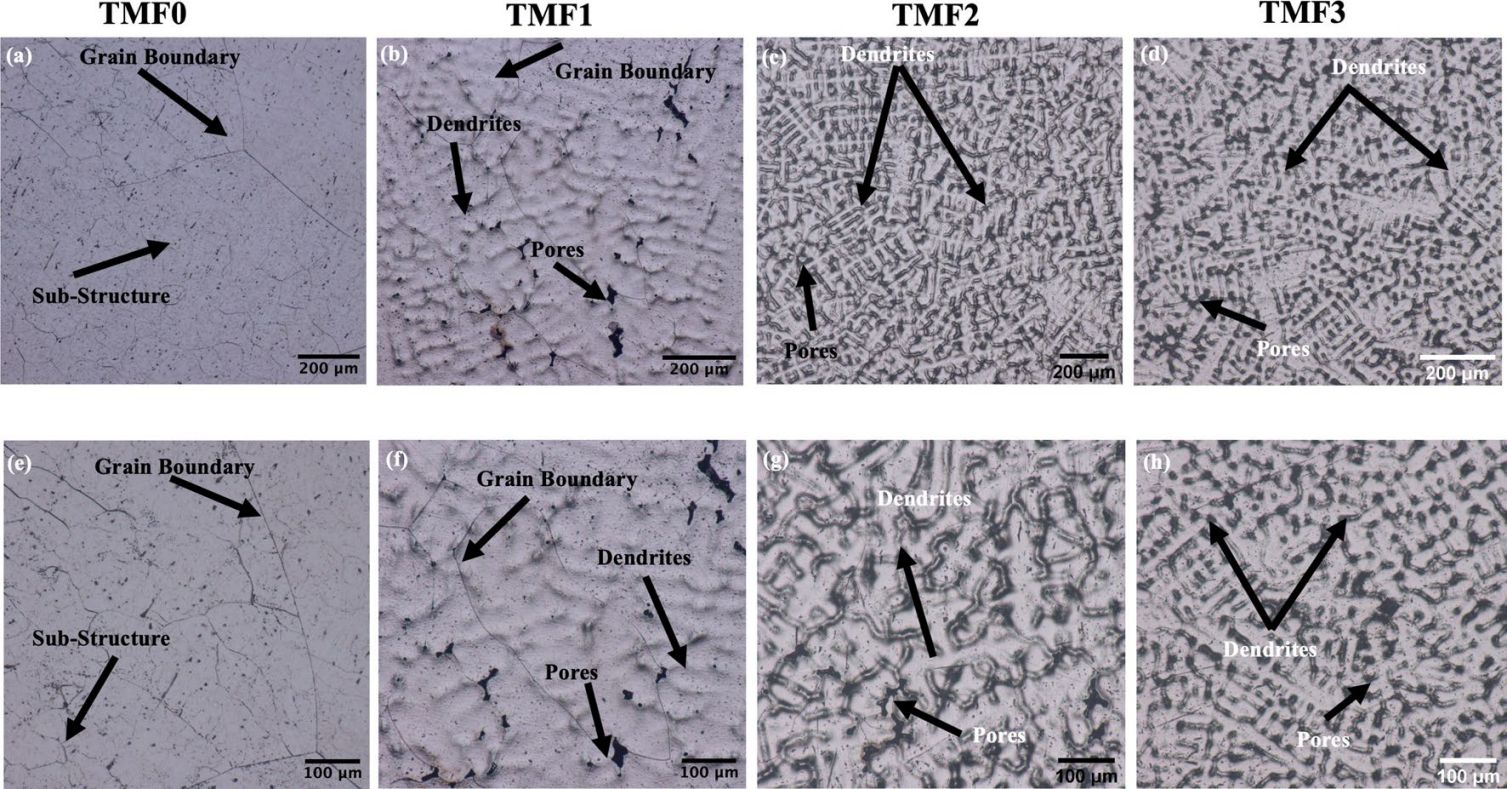
Optical micrographs of as-cast TMF0, TMF1, TMF2 and TMF3 alloys in low and high magnifications.

**Figure 4:**
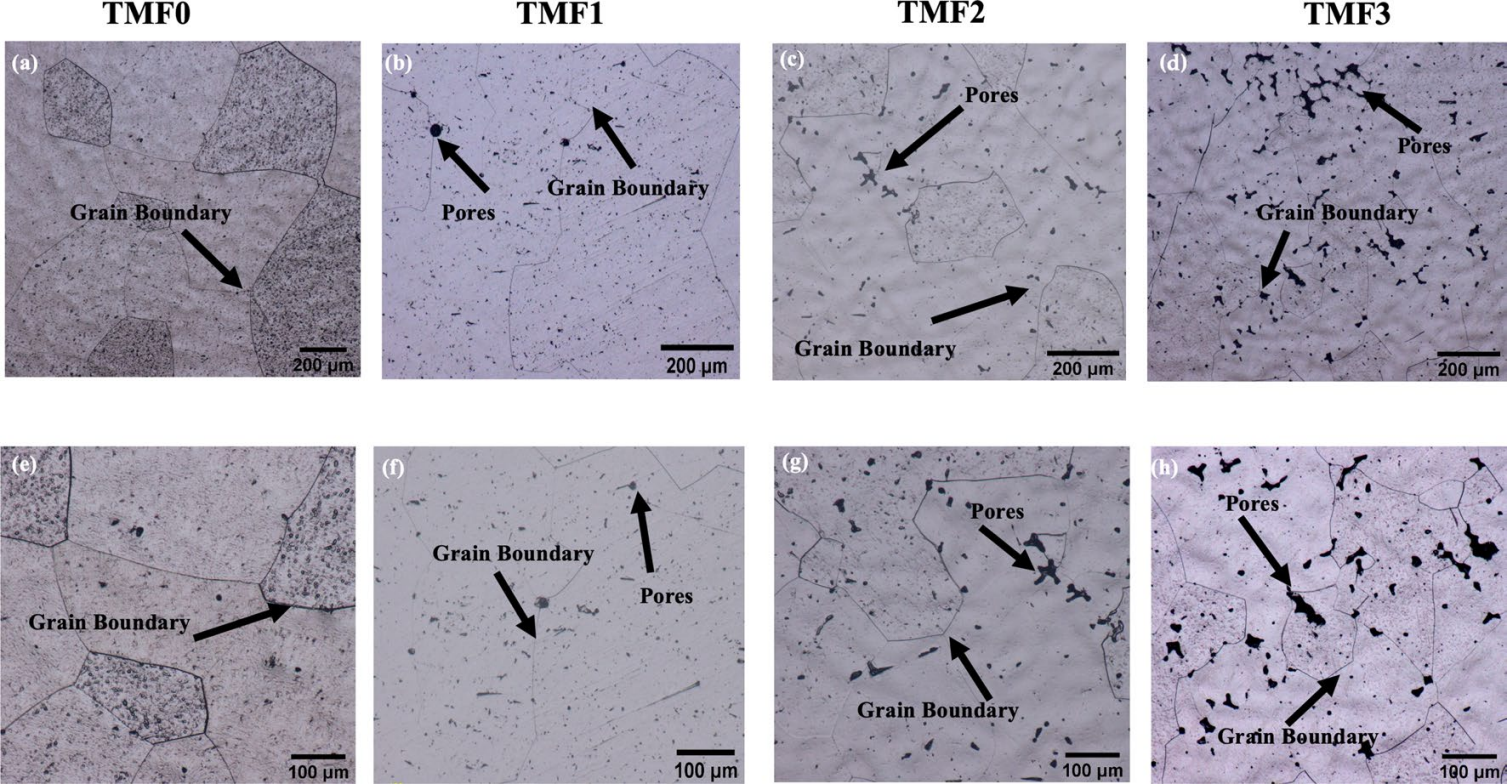
Optical micrographs of water quenched TMF0, TMF1, TMF2 and TMF3 alloys in low and high magnification.

The optical micrographs of the as-cast and quenched samples are presented in Fig. 3. It is observered that the as-cast TMF0 alloy in Fig. 3a, b depicted large grains of equiaxed β phase, with high volume fraction of sub-grain structures existing within the large grains. The observered sub-grains may possibly be associated with the presence of the orthorhombic martensitic α′′ phase. The α′′ is an intermediate phase during the β (BCC) to α (HCP) transformation, and it requires smaller strains to form compared to the equilibrium α phase. Such strains can be induced either thermally through non-equilibrium cooling or by mechanically means^26^. As shown later, the XRD analysis was carried out to confirm the presence of martensitic α′′ phase. The presence of the α′′ or the sub-grain structure could signify that the cooling medium during melting was not fast enough to stabilize the β phase only but was fast enough to induce sub-grain structures. When TMF0 was solution treated and quenched in ice-brine, the micrograph (Fig. 4a, e) was composed of only β equiaxed grains with no additional phases present. The grains were smaller in size as compared to those in the as-cast condition. The presence of the small equiaxed grains showed that the cooling rate during quenching was high enough to stabilize the β phase and impede the transformation to α′′ phase as compared to the cooling rate during casting. This will also be verified by the use of XRD analysis. The addition of 4 wt.% of Fe into Ti–15Mo alloy, that is TMF1, resulted in the presence of dendritic structure within the β equiaxed grains and along the grain boundaries in the as-cast condition, as shown in Fig. 3b, c. This micrograph also revealed the presence of pores around the grain boundaries and pitting that occurred during the etching process. However, the OM micrographs of quenched sample presented in Fig. 4b, f illustrated only equiaxed grains of β phase without any dendritic structures. The micrographs also showed pores around the grain boundaries and a higher finely distributed pitting as compared to the as-cast sample. At much higher Fe content, 8 and 12 wt.% here referred ad TMF2 and TMF3 respectively, the micrographs for the as-cast condition reveal a structure comprised completely of inhomogeneous dendrites without any observable grain boundaries, as shown in Fig. 3c, e and d, f. The corresponding micrographs for the quenched samples (Fig. 4c, d) reveal only equiaxed grains of β phase with pores around the grain boundaries. The occurrence of dendritic structure is due to rapid solidification process as a result of the very fast movement of the liquid/solid interface toward the undercooled melt^27^. One typical feature on the microstructure of various Ti alloys is the dendritic structure due to the ability to generate dendritic patterns^28^. The presence of dendritic structure could be influenced by several factors such as cooling rate and alloy composition, for instance a finer and coarser dendritic structure could be produced by rapid cooling^29^. The studied OM micrographs of TMF0 is consistent to the one reported by Moshokoa et al. 2021, due to less work reported on TMF1, TMF2 and TMF3, these alloys can be comparable to other published Ti based alloys. For instance a completely dendritic structure in TMF2 and TMF3 was found to be comparable to dendritic structure of NiTi alloy in cast condition investigated by^30^. Okulov et al. also reported the presence of dendritic structure on the Ti–13.6Nb–6.5Al–6Cu–5.1Ni and Ti–14.1Nb–6.7Al–4Cu–3.4Ni alloys in cast conditions^21^. The dendritic structure in TMF1, TMF2 and TMF3 in as-cast condition are comparable to the ones in Ti–19Fe–5Sn–1Mo, Ti–17Fe–5Sn–3Mo, and Ti–15Fe–5Sn–5Mo alloy reported by^23^.

### Micro-vickers hardness

**Figure 5:**
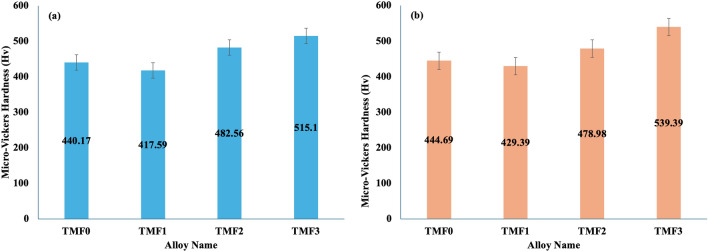
Micro-Vickers Hardness of (**a**) as-cast and (**b**) quenched TMF alloys.

Hardness should be high enough to avoid shear failure during biomedical or refractory performance^31,32^. Figure 5a, b presents hardness values of as-cast and quenched samples, respectively, which resulted in almost similar trends. The hardness of TMF0 was found to be 440.17 Hv_0.5_ in the as-cast condition, the hardness increased slightly to 444.69 Hv_0.5_ upon quenching. The addition of 4 wt.%, resulted in the hardness value of 417.59 Hv_0.5_ in the as-cast condition and its hardness increased to 429.39 Hv_0.5_ after quenching. A further increase in the Fe content to 8 wt.% yielded a hardness of 482.56 Hv_0.5_ in the as-cast condition but this hardness decreased slightly to 478.98 Hv_0.5_ when subjected to quenching. The TMF3 alloy with 12 wt.% Fe content was measured to have a hardness of 515.10 Hv_0.5_ in the as-cast condition. A significant increase in hardness to 539.39 Hv_0.5_ was observed upon quenching. The hardness of different Ti-alloy phases are summarized in the following increasing order H_α″_ < H_β_ < H_α_ < H_α'_ < H_ω_ according to^33^. The above trend entails that omega phase (ω) has the highest hardness, followed by HCP α' phase which has a hardness value that is more than that of α phase. The β phase has hardness only higher than that of α″ phase. However, the following hardness trend reported by Lee et al.^34^ is slightly different H_ω_ > H_α'_ > H_α″_ > H_β_ > H_α_. This trend implies that the hardness of α″ phase is higher than that of β phase whereas the α phase is the least hard.

The hardness results reported in the current work research is in agreement with the hardness trend reported by^34^. Our observation is that the hardness of the quenched TMF0 alloy comprised of 100% β phase is higher than that in the as-cast condition. This higher hardness is mainly attributed to the finer grains as observed on the micrographs of the quenched sample in Fig. 4f compared to that of the as-cast in Fig. 3a. The increase in the hardness in TMF1 after quenching may be due to higher volume fraction of orthorhombic martensitic α″ phase compared to in the as-cast condition. High hardness of TMF2 in the as-cast condition could be due to several factors such as: the presence of high volume fraction of the brittle intermetallic B2 TiFe phase, the orthorhombic martensitic α″ phase as shown in the XRD patterns in Fig. 1c, as well as a full dendritic structure observered in the OM micrographs in Fig. 3c. In addition, high Fe content promotes solid solution strengthening effect. The slight decrease in the hardness after quenching may be attributed to the absence of α″ phase. It has been reported that the presence of porosity reduces the elastic modulus of a component, however, it can also lead to local stress concentrations which decrease the strength, hardness, and ductility of the alloys^35^ In comparison to the sample in the as-cast condition, a significant increase in hardness is observed in the TMF3 alloy upon quenching. Since the solid solution strengthening effect is common in both samples containing high Fe, it is highly possible that rapid oxidation of the intermetallic B2 phase could be responsible for increased hardness. It is for this reason that all the ternary samples were found to be very brittle, suggesting that the intermetallic B2 phase to be most prone to oxidation than the β matrix. The hardness of as-cast TMF0 alloy is found to be higher compared to those reported in^36^ for Ti–15Mo alloy (307 HV_0.2_) and ^36^ for Ti–15Mo alloy (330 HV_0.5_) in the as-cast condition. The micro-Vickers hardness of the studied alloys was compared to other metastable alloys found in the literature. The TMF2 and TMF3 alloys were higher as compared to Ti–10Ta–4Fe (410Hv), Ti–12Nb–5Fe (293Hv), Ti–7Ta–5Fe (430Hv) alloys, while the TMF1 was lower than Ti–7Ta–5Fe alloy. The micro-Vickers hardness of the studied alloys was higher than the commercially pure (CP) Ti (156 HV_0.2_) reported by^36^ and 210 HV_0.5_ in CP-Ti alloy^37^.

### Tensile properties

Tensile properties were measured using the tensile tester machine. Tensile properties such as ultimate tensile, elastic modulus were recorded. The elastic modulus or stiffness should be as close as possible to that of the human bone to avoid stress shielding effect in orthopedic implants^31,32^. TMF0 alloy depicted an elastic modulus of 79 GPa in the as-cast condition while the elastic modulus upon quenching was measured to be 66 GPa. The elastic’s modulus (*E*), which is an intrinsic materials property, depends on atomic bonding force amongst atoms and the bonding force is not only related to the crystal structure but also to the distances between atoms. Thus *E* can be affected by the atomic radius of the alloying element, heat treatment process and plastic deformation^38^ and^39^. Any change in the distance between atoms of the material leads to variation in the atomic bonding force, and thus affect the resulting elastic modulus. The most common phenomenon is that a phase change caused by a heat treatment or stress may alter the elastic modulus of a metallic material because it changes the distance between atoms, which is why the different phases (crystal structures) have different elastic moduli^40^. Thus, the significant decrease in the elastic modulus of TMF0 after quenching may be attributed to the absence of the α″ phase which has higher elastic modulus than the β phase. Higher elastic modulus in the as-cast condition may be due to presence of the orthorhombic martensitic phase α″ as observered in the XRD patterns. It is widely reported that the β-Ti alloys which retains 100% β phase possess lower elastic modulus than those containing α, α', α″ and ω phases^41^. This is in accordance with the elastic modulus trend reported by^42^ which indicates β phase have the least elastic modulus. The elastic modulus of TMF1 alloy in the as-cast condition was measured to be 74 GPa which is 5 GPa lower than that of TMF0 alloy. The slight decrease in the elastic modulus is despite the presence of the hard and brittle B2 intermetallic phase.

Mechanical strength is vital property in structural applications or load bearing applications as it is required to maintain support. Similarly, the implants used under load need to possess much higher strength than the stress they will experience during service^43^. The measured ultimate tensile strength (UTS) of TMF0 in the as-cast condition is 264 MPa. However, upon quenching, this value almost doubled to 411 MPa. The UTS of TMF1, TMF2 and TMF3 in the as-cast and quenched conditions could not be measured due to the brittleness of the material, the samples crumbled or broke before they could reach a yield point. This high level of fragility is owed to the presence of most brittle B2 TiFe and its oxidised phases in the as-cast and quenched conditions, respectively. The tensile properties (UTS and elastic modulus) of TMF0 alloy in quenched condition are much lower than 594 MPa and 70.5 GPa, respectively, reported by^19^. The reason for this high modulus in studied alloy could be attributed by the presence of pores precipitated along the grain boundaries and inside the grains. The percentage porosity or the presence of pores were not reported by Moshokoa et al. in their published work. The tensile strength of the TMF0 alloy in the as-cast and quenched conditions was found to be significantly lower compared to the commercially used Ti6Al4V alloy (825-895 MPa). Similarly, the elastic modulus of both TMF0 and TMF1 in the as-cast condition were lower than that of Ti6Al4V alloy (111GPa)^44^. The elastic modulus of TMF1 in the as-cast condition was even lower than that of Ti–12Nb–5Fe (90 GPa)^24^, Ti–19Nb–2.5Fe (90GPa)^45^ and Ti–30Nb–3Fe (81 GPa)^46^. The above tensile results show that TMF0 in quenched condition shows better properties to be used as a potential alloy for the manufacturing of orthopedic implants, however the ternary alloys illustrated minimal possibility because of their brittleness that was attributed to the presence of intermetallic B2 phase in the as-cast condition or their resulting oxide upon heat treatment.

### Compression properties

**Figure 6:**
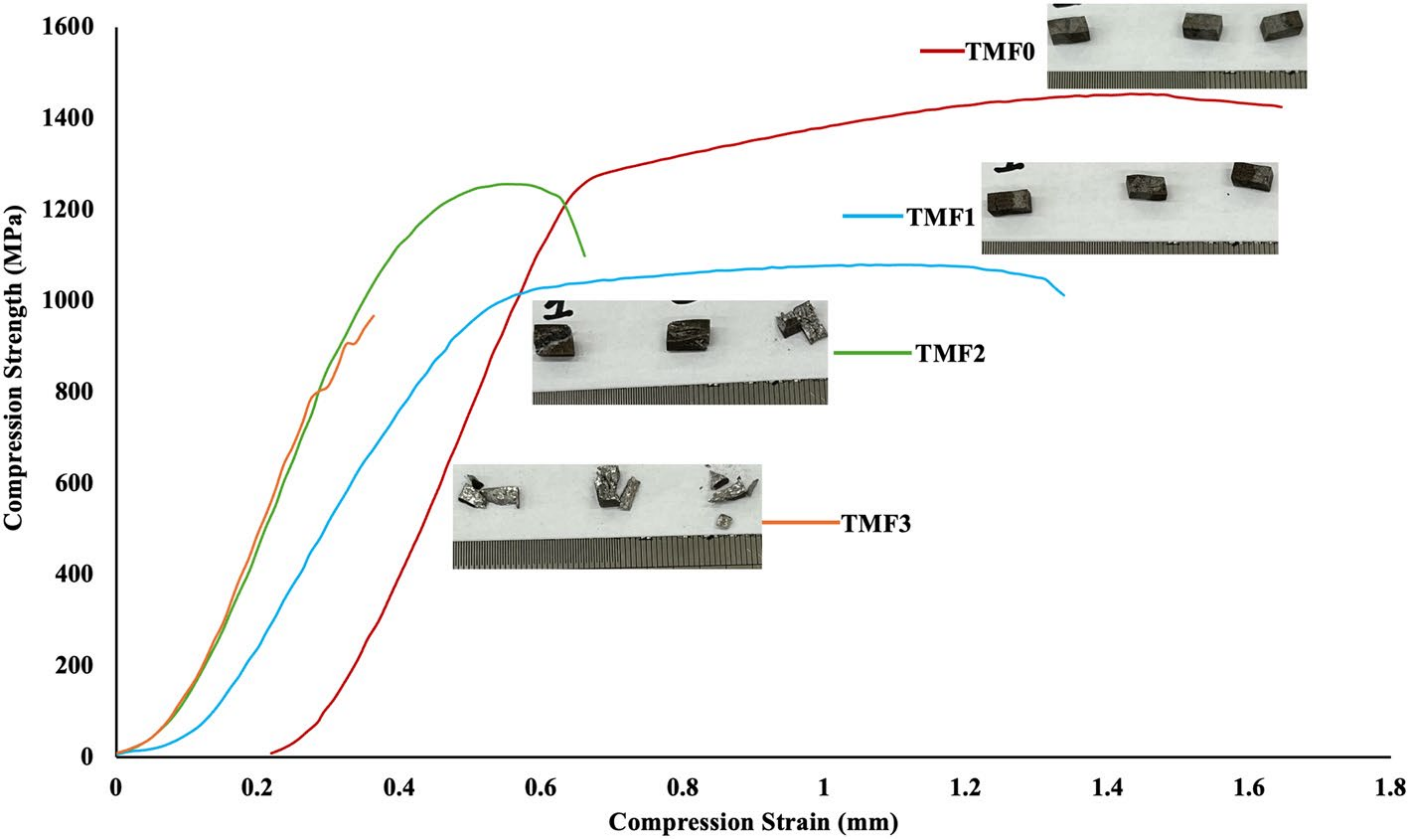
Compression stress–strain curve of As-cast alloys.

**Figure 7:**
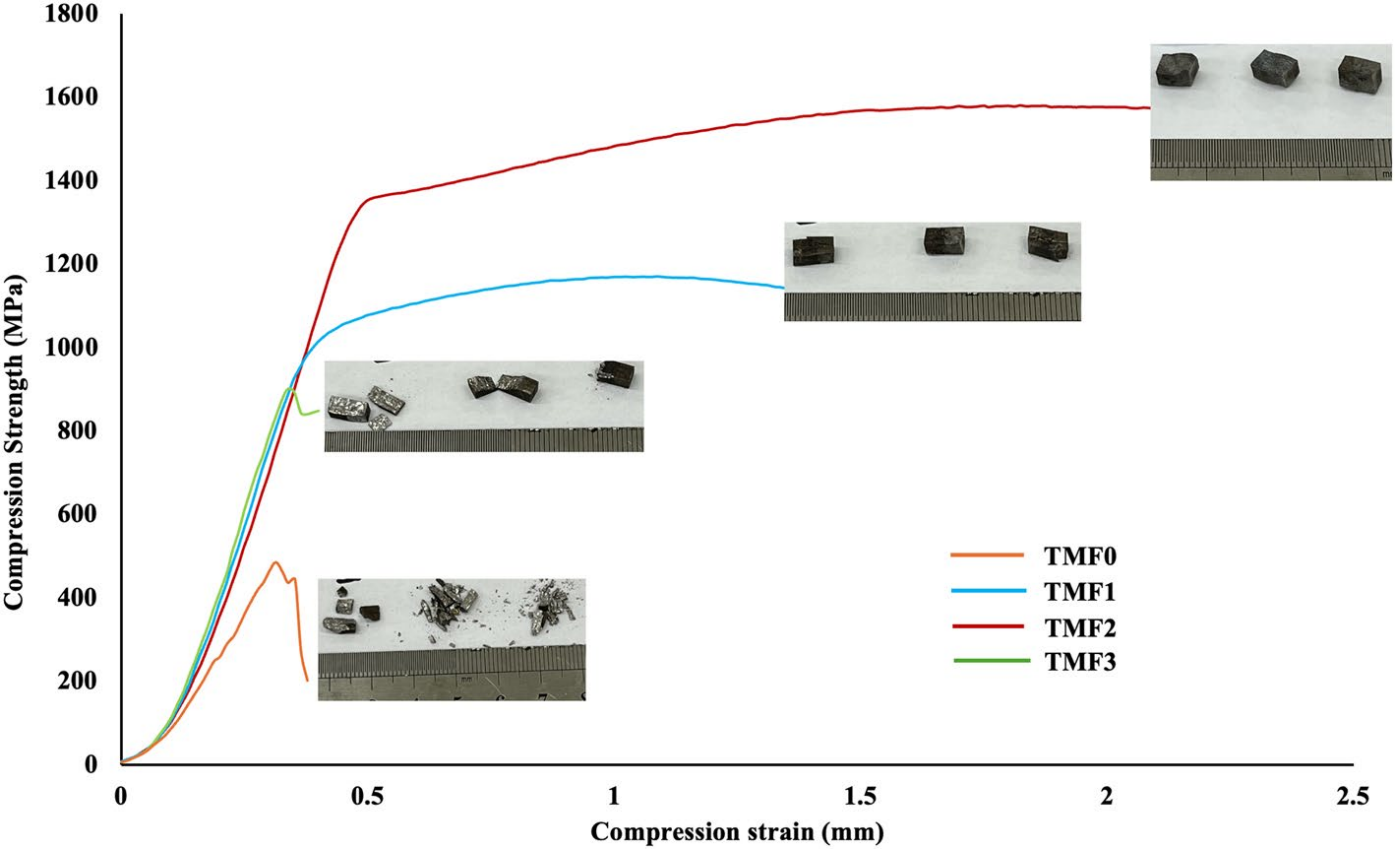
Compression stress–strain curve of water quenched alloys.

Figures 6 and 7 present room temperature compressive stress vs strain curve of the as-cast and quenched alloys. According to literature, a compressive stress–strain curve comprises of 3 stages: stage I, elastic deformation, stage II, a plastic yield plateau in which the stress increases very slowly linearly with strain, and stage III, a parabolic stage. According to Figs. 6 and 7 TMF0 and TMF1 alloys exhibited all the three stages, while TMF2 and TMF3 showed only stage one which was the elastic deformation stage. The brittleness of the samples of TMF2 and TMF3 caused early failure of the samples. TMF0 showed great work hardening as compared to the other alloys and this could be by the present of the dominant β phase after quenching and the decreased in the work hardening in TMF1 could be attributed by presence of the more brittle of mixed oxide Fe·Ti·O_2_ that transformed from the B2 intermetallic phase (TiFe) after quenching. The TMF2 and TMF3 suffered a great deal in terms of hardening after quenching, the samples broke during testing before they could reach the maximum testing load that was set. With the high content of Fe the compression strength was expected to be high as Fe is a solid solution strengthening element, however due to the brittleness of the intermetallic phase (TiFe) that transformant into a more brittle mixed oxide of Fe·Ti·O_2_ , the samples failed before reaching their maximum load. This shows that the effect of the B2 intermetallic phase in as-cast and the brittle mixed oxide Fe·Ti·O_2_ have a significant effect on the mechanical properties and they should be avoided at all cost. Similar compressive stress -strain curves have been reported by^47^ in Ti–22.4Nb–0.73Ta–2Zr–1.34O, Ti–40Ta–22Hf–11.7Zr and Ti–45Ta–18.4Hf–10Zr^48^. TMF0 fractured at 1.8% strain while the TMF1 around 1.4% strain and the TMF2 and TMF3 fractured at lower strain of 0,07%. TMF0 and TMF1 alloys have enhanced the plastic deformation ability as compared to TMF2 and TMF3 alloys and they are the ones that shows the ability to be considered for biomedical applications.

**Figure 8:**
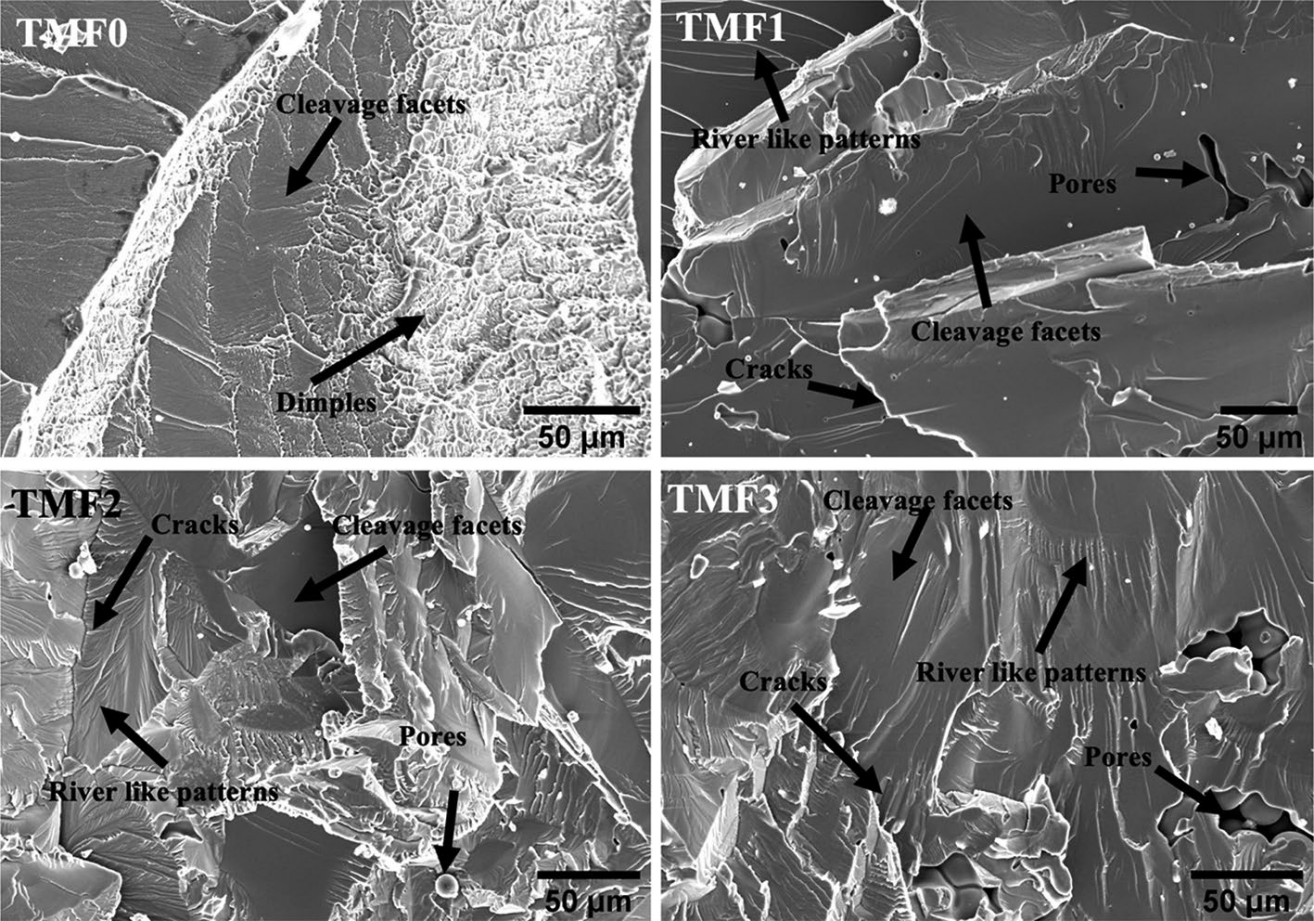
Fracture surface of as-cast alloys after tensile test.

The compression properties such as the ultimate compression strength, yield strength and the elongation in as-cast and quenched conditions are presented in Tables 1 and 2 respectively. The as-cast TMF0 illustrated an ultimate compression strength of 1396.67 MPa and it increased significantly to 1568.93 MPa after quenching in icy water. TMF1 in as-cast condition exhibited an ultimate strength of 1037.5 MPa and it notably increased to 2780.4 MPa after quenching. The TMF2 in as-cast showed the ultimate strength of 1156.27 which decreased greatly to 483.38 MPa after quenching. The TMF3 showed an ultimate strength of 878.6 MPa in as-cast condition and creased by almost half (404.6 MPa) after quenching. The compressive yield strength increased slightly after quenching from 1279.96 MPa in as-cast to 1300.64 MPa and the that of TMF1 was 921.22 MPa in as-cast and 841.91 MPa in as quenched. The TMF2 and TMF3 alloys did not exhibit any yield strength as the samples were to brittle to yield under the applied load. The compression extension decreased with the addition of Fe in both as-cast and quenched conditions. Despite the solid strengthening mechanism of Fe, the decrease in the strengths and the elongation in as-cast condition as the Fe contend increased could be attributed by the presence of the brittle B2 intermetallic phase (TiFe) as observered in the XRD and OM micrographs. As it can observed from the stress–strain graphs in in Figs. 7 and 8, the TMF2 and TMF3 are the most brittle as they could not resist fracture before reaching their maximum load. The decrease in strengths and elongation after quenching with the addition of Fe could be attributed to the presence of the brittle mixed oxides Fe·Ti·O_2_ as observered in the XRD patterns. The above results shows that TMF0 and TMF1 are the promising alloys that can be used in biomedical applications, while TMF2 and TMF3 could be considered as materials for other applications.

**Table 1:** Compression properties of as-cast alloys.

Designated name	Ultimate compression strength (MPa)	Compressive Yield strength (MPa)	Compression elongation (%)
TMF-0	1396.67	1279.96	0.99
TMF-1	1037.5	921.22	0.85
TMF-2	1156.27	–	0.48
TMF-3	878.6	–	0.35

**Table 2:** Compression properties of quenched alloys.

Designated alloy name	Ultimate compression strength (MPa)	Compressive yield strength (MPa)	Compression elongation (%)
TMF-0	1568.93	1300.04	1.803
TMF-1	2780.4	841.91	0.650
TMF-2	483.38	–	0.372
TMF-3	404.6	–	0.313

### Fracture surfaces analysis

**Figure 9:**
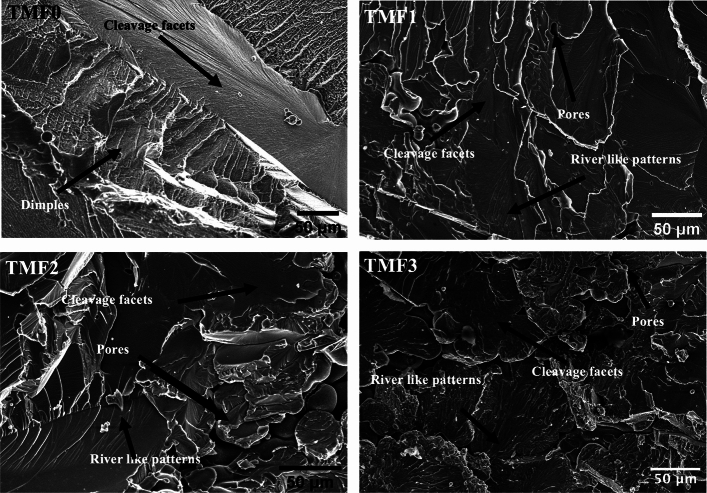
Fracture surface of quenched alloys.

Fractography analysis of tensile specimens after tensile test were carried out on both the as-cast and quenched samples. SEM micrographs of fracture surfaces of tensile specimens are depicted in Figs. 8 and 9 for the samples in the as-cast and quenched conditions, respectively. As shown in Figs. 8 and 9, the TMF0 in the as-cast and quenched conditions exhibits both ductile and brittle fracture. The ductile fracture is characterized by dimple like fracture, while brittle fracture can be seen by cleavage fracture where river like patterns can be seen on the fracture surface. As shown in Fig. 8, the fracture surfaces of TMF1, TMF2 and TMF3 samples in the as-cast condition comprises of only the brittle fracture. The same applies for TMF1, TMF2, and TMF3 samples in the quenched condition, as shown in Fig. 9. The micrographs were composed of river like patterns on the fracture surfaces, pores, cleavage facets and cracks that were growing along the fracture surfaces. The river like patterns, the cleavage facets and pores increased in fraction with an increase in the Fe content. The presence of pores at high Fe content could be attributed to shorter melting time as they were flipped only 3 times only. The increase in the melting time could eliminate their presence as they will have longer time to melt. The brittle fracture in the as-cast samples can be attributed to the precipitation of brittle intermetallic B2 TiFe phase as confirmed by the XRD analysis. On the other hand, the brittle fracture in the quenched samples is as a result of finely distributed brittle metal oxide formed from oxidation of intermetallic B2 phase. Moreover, it is reported in literature that a higher addition of a strengthening element leads to beta flecks or TiFe intermetallic that have adverse effects on the me chanical properties^16^.

## Conclusions

Microstructural characteristics, porosity evaluation, phase analysis, and tensile properties along with hardness results of the as-cast and quenched Ti–15Mo–xFe (x = 0, 4, 8, 12 wt%) alloys were systematically investigated for orthopedic applications. The following conclusions were drawn on the basis of the results of the study:

1. The OM micrographs of TMF0 in as cast condition demonstrated large equiaxed β structure and sub-structures of α′′ phase, while the quenched sample was comprised of only finer equiaxed β grains. The micrographs of TMF1, TMF2 and TMF3 illustrated dendritic structure and pores in the as-cast condition whereas micrographs of their quenched samples depicted only large equiaxed grains of β without the dendrites.
2. The XRD analysis conformed that TMF0 in as-cast condition comprised of both bcc β and orthorhombic martensitic α′′ phases, whereas the quenched alloy was composed of only β phase. TMF1, TMF2, TMF3 in as-cast condition encompassed the formation of an intermetallic phase of TiFe and their quenched alloys were contained the β phase, α′′ phase, and a mixed oxide layer.
3. The Micro-Hardness of the studied alloys showed similar trend in both as-cast and quenched conditions, where the hardness increased slightly in quenched samples.
4. The elastic modulus of TMF0 decreased drastically after quenching. Comparing the elastic modulus of TMF0 and TMF1 in as-cast condition, the TMF1 showed the lowest modulus as compared to TMF0. Most tensile properties of TMF2, and TMF3 in as-cast and quenching conditions could not be determined due to the fragility of the samples.
5. Compression properties showed that the addition of Fe decreased both yield and ultimate strength along with the elongation. Heat treatment improved the properties, however due to fragility and high content of Fe TMF2 and TMF3 with high content of intermetallic phase were too brittle to yield any results.
6. The fracture surfaces of TMF0 exhibited both ductile and brittle fracture in as-cast and quenched conditions whereas the only brittle fracture mode was observed in the TMF1, TMF2 and TMF3 alloys in both conditions.

In summary, a concept of evaluating the effect of high addition of Fe content on Ti–15Mo alloy for orthopedic application was realised. It was discovered that the higher the content of Fe into Ti–15Mo alloy results in a formation of a brittle intermetallic B2 TiFe phase which negatively affect the mechanical properties. The tensile properties of alloys with high content of Fe could not be recorded due to the fragility of the samples. The compression properties showed that as Fe increases the more brittle the material became due to the formation of B2 TiFe phase and how the intermetallic phase affected the strength. Thus the studied ternary alloys especially TMF2 and TMF3 cannot ideal for orthopedic application due to the high brittleness. The studied alloys can be considered for other application but for surface coatings the designed alloy should be biocompatible, have high strength, have high wear and corrosion resistance. The alloys meet some of the requirements such as biocompatibility, high wear due to the high hardness, however the presence of the brittle TiFe in as-cast condition and brittle mixed oxides of Fe·Ti·O_2_ makes them less candidate materials for surface coatings.Ti-35.

## Data Availability

The data that support the findings of this study are available from the corresponding author (Moshokoa Nthabiseng and Maje Phasha) upon request.
